# Exploring the relationship between exposure to COVID-19 and donations during the COVID-19 pandemic: The mediating roles of emotions and risk perception

**DOI:** 10.3389/fpsyg.2023.1052531

**Published:** 2023-04-04

**Authors:** Yun Bao, Yan Zhang, Junxiu Wang

**Affiliations:** ^1^Department of Psychology, New York University, New York, NY, United States; ^2^The Affiliated Kangning Hospital of Wenzhou Medical University, Wenzhou, Zhejiang, China; ^3^Institute of Sociology, Chinese Academy of Social Sciences, Beijing, China

**Keywords:** donation, COVID-19 pandemic, terror management theory, risk perception, emotion, public emergency

## Abstract

**Objective:**

Public emergency events like the COVID-19 pandemic are special occasions that need immediate massive funding from public donations. Thus, understanding the determinants of donation behaviors under public emergencies is important for both researchers and practitioners. This study investigated the effect of personal and local exposure to incidences of COVID-19 on donation behaviors. Specifically, we examined the mediating effects of risk perception and emotions on the relationship between exposure to COVID-19 and donation behaviors.

**Methods:**

The data were from a survey distributed in China between March 20 and 30th, 2020. Participants’ donation choice at the end of the survey was used to measure their donation behaviors. Participants’ emotions, risk perception, and personal exposure were assessed in the questionnaire. Local exposure was the 30-day confirmed cases obtained from the National Health Commission of the People’s Republic of China. A total of 8,720 participants (Mean age = 28.91, 43.6% females) completed the online survey.

**Results:**

Based on the results from the mediation analysis, we found that people with stronger positive and negative emotions, higher risk perception, and more personal exposure to COVID-19 were more likely to donate. Furthermore, the effects of both personal and local exposure on donations are mediated by risk perception and negative emotion. Both higher personal and local exposure led to stronger negative emotions and higher risk perception, which in turn led to more donation behaviors.

**Discussion:**

This study extends our knowledge of donation behaviors during public emergencies. Our results suggest that policymakers and charity organizations should elicit stronger emotions and risk perception by exposing the severity of the disaster in advertisements to promote donations.

## 1. Introduction

The novel coronavirus disease 2019 (COVID-19) pandemic is the most recent public health emergency that has caused a devastating impact across the globe. Since its first onset in late 2019, COVID-19 has led to 6.22 million deaths worldwide during the past 3 years (Center for Systems Science and Engineering at Johns Hopkins University, 2022). During the pandemic, government funding alone is not enough to overcome the healthcare system burden, the massive unemployment, the financial difficulty of hospitality industries and other corporations, and the lack of living necessities (Baber et al., 2022). Donation from the general public plays an important role in mitigating the pressure in different social aspects. Yet, in China, the monetary donation from the individual level was relatively low compared to donations from other social sectors. For instance, China Charity Alliance (CCA) reported that, until February 3rd, 2020, among the 16.167 billion Chinese Yuan total donation for pandemic control in China, the individual-level donation was 227 million Chinese Yuan, which was only 1.4% of the total amount (China Charity Alliance, 2020). Thus, understanding the conditions in which people donate more is important to promote individual donations during public emergencies.

Past research has generated some meaningful findings on people’s donation behaviors in the context of natural disasters, for instance, hurricanes, earthquakes, and floods (Destro and Holguín-Veras, 2011; Korolov et al., 2016; Utomo et al., 2020) but public health events have not been extensively explored (Zagefka and James, 2015). As a representative public health emergency of international concern, the COVID-19 pandemic provides a new challenging social context for psychological research on individual donation behaviors. On the individual level, the COVID-19 pandemic put people under a collective threat due to its high transmissibility and mortality, drastically increasing people’s risk perception and fear of death. In addition, reduced social connections and the threats of reduced financial income also generated severe stress, need dissatisfaction, and uncertainty among individuals (Han et al., 2021; Zhang and Wang, 2022). However, on a population level, because of their shared experience with the COVID-19 pandemic, people might perceive all human beings as a community with a shared future, which might alter their donation behaviors compared to normal times. Under this social context, how would individuals’ experiences with COVID-19 shape their risk perception and emotional states? How would these psychological states influence their donation behaviors?

Recent studies have examined the effect of exposure to COVID-19 on people’s donation behavior (Blanco et al., 2021; Grimalda et al., 2021; Zheng et al., 2021b). However, most research did not separate personal exposure and local exposure, which are two different kinds of experiences for individuals. Personal exposure refers to one or one’s close relatives and friends having experiences of being diagnosed with COVID-19; Local exposure refers to the existence of the pandemic in the area in which one resides. Would people personally exposed to COVID-19 have a different donation pattern from those who are only locally exposed to it? Besides, most relevant research done in western countries (McGuire et al., 2020; Blanco et al., 2021; Grimalda et al., 2021; Jin and Ryu, 2021; Zheng et al., 2021b) has opposite results from research done in China (Zheng et al., 2021a; Li W. Q. et al., 2021; Li S. et al., 2021; Lee et al., 2021). Local exposure to COVID-19 was found to have a significant positive effect on donations in most western countries but not in China. Whether the research findings in western countries can hold in the social context of China is still questionable.

To answer these questions and expand the existing literature, we used the 2020 COVID-19 pandemic in China as the background to examine the psychological mechanism of individual donation patterns under public emergencies. Unlike other COVID-19-related research, this study separated personal and local exposure, exploring the effects of subjective individual experience and objective societal situations on donations. In addition, by integrating Terror Management Theory and risk perception theories, we investigated the psychological mechanism of how exposure to COVID-19 motivated people to donate more or less *via* its emotional consequences and the cognitive evaluation it led people to possess. Specifically, the current study explored the effects of personal and local exposure on donation behaviors as well as the mediating effects of risk perception and emotions on this relationship.

## 2. Theoretical background and hypothesis development

**Figure 1:**
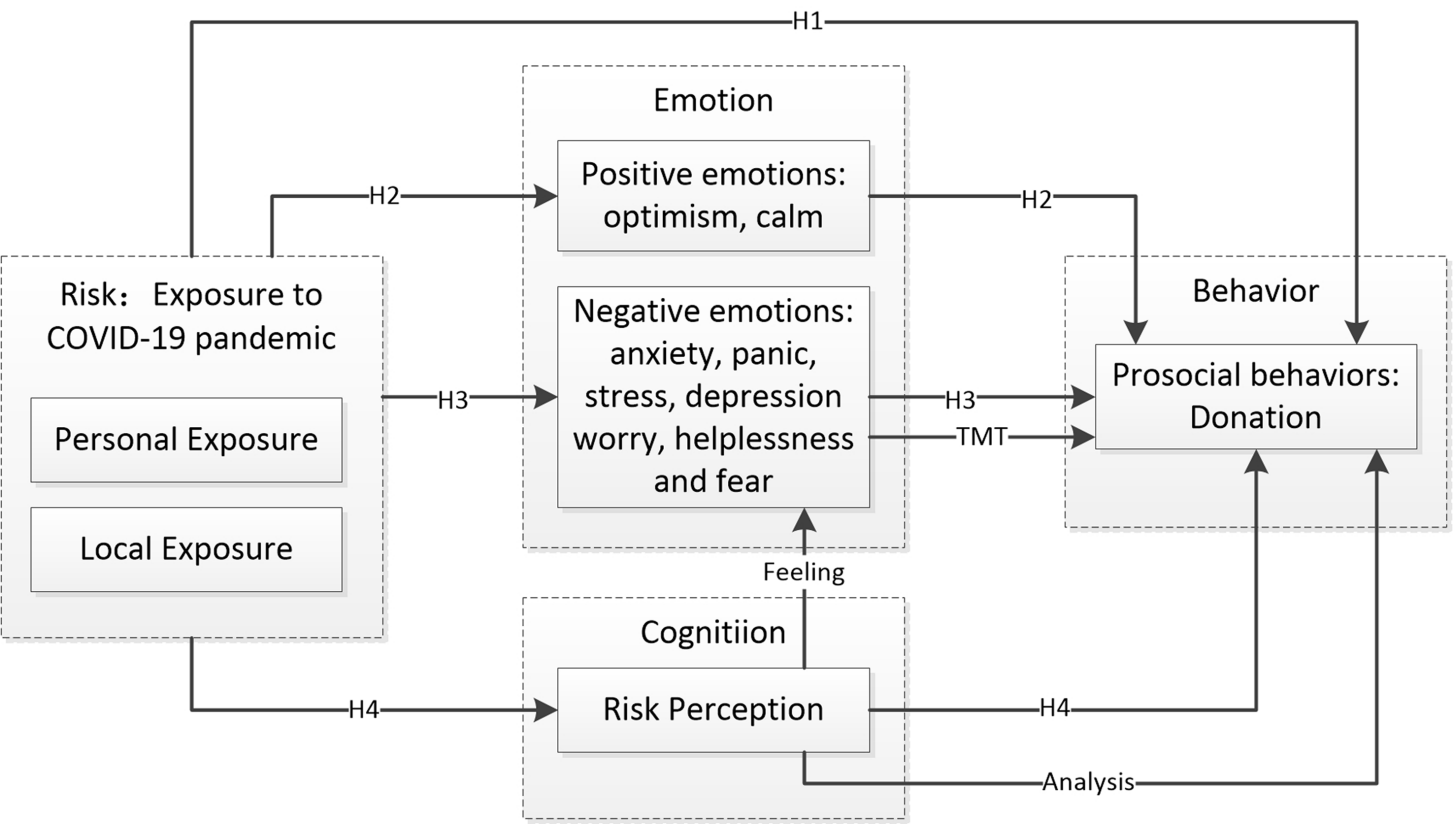
A psychological framework and hypotheses of donation behaviors in the context of COVID-19 pandemic.

From a psychological perspective, the determinants of donation are primarily based on emotional states (affective mechanisms) and rational processes (cognitive mechanisms; Dickert et al., 2011a,b). Thus, we proposed that emotions and risk perception are the two main factors that explain the motivational effect of exposure to COVID-19 on donation behaviors. Drawing on available theories on emotions, risk perceptions, and donation, we developed and proposed a psychological framework for donations in the context of the COVID-19 pandemic (Figure 1). In the present framework, personal exposure and local exposure to COVID-19 are antecedents that influence donation behaviors, while emotions and risk perception are considered as possible mediators of the relationship between exposure to COVID-19 and donation behaviors. On this basis, a set of hypotheses is developed.

### 2.1. The impact of exposure to COVID-19 on donation behaviors

In early 2020, without an effective vaccine, the impact of the COVID-19 pandemic was under high uncertainty due to its high infection rate and mortality (Ruiu, 2020), which significantly affected people’s cognition, emotions, and behaviors. Under life-threatening crises such as wars, epidemics, and natural disasters, people tend to have a paradoxical reaction to the situation, such that they might be more selfish and more altruistic simultaneously (Fridman et al., 2022). On the one hand, evidence has shown that threats and uncertainty would decrease one’s willingness to participate in charity activities (Poulin et al., 2012). On the other hand, groups of people present stronger social cohesion (Gilligan et al., 2014) and more cooperative behaviors when facing collective threats (Kaniasty and Norris, 1995; Gneezy and Fessler, 2012; Li et al., 2013). As Zaki (2020) proposed in his Catastrophe Compassion Theory, disasters may increase people’s awareness of community and altruism. Therefore, facing the COVID-19 pandemic, people may show contradictory patterns of prosocial behaviors.

In fact, current research conducted in the United States, Italy, United Kingdom, Spain, China, and other countries studying people’s donation behaviors under COVID-19 has shown this complex antithetical situation. A great portion of research concludes that personal experience with the pandemic and the severity of the pandemic in the local area positively affect donation willingness (Zhang and Meng, 2021; Zheng et al., 2021b; Adena and Harke, 2022). For example, Grimalda et al. (2021) showed that people were more likely to donate if their relatives, acquaintances, or themselves had been diagnosed with COVID-19. As for local exposure, research has also shown that the number of newly confirmed cases of COVID-19 in the local community has a significant positive relationship with donations (Blanco et al., 2021). Outside the scope of the pandemic, a similar relationship was also observed in general donations and natural disasters. Research has found that physical proximity to people in need or the area impacted by disasters is positively associated with monetary donations (Destro and Holguín-Veras, 2011; Zagefka and James, 2015; Touré-Tillery and Fishbach, 2017). Thus, if the participants are closer to the area where the pandemic exists, they tend to donate more.

However, some research discovered that local exposure to the pandemic has no significant influence or negative effect on donation behavior (Zheng et al., 2021a). In the study of Grimalda et al. (2021), although the positive correlation between personal exposure and donation behaviors was confirmed, local-confirmed cases did not affect donation behaviors. As research of Brañas-Garza et al. (2022) showed, the severity of the pandemic has no significant influence on young people’s donation behavior but a negative influence on older people’s donation behavior. Fridman et al. (2022) also showed that the existence of COVID-19 in the local community could significantly promote donations, while the severity of the local pandemic has no significant effect. Since nearly the same amount of literature supported either side of the story, we had no prior expectations regarding the direction of local exposure’s overall effect on donations and proposed the following open hypothesis:

H1: Both personal exposure and local exposure to COVID-19 influence people’s donation behaviors.

a: Personal exposure to COVID-19 has a significant positive effect on donation behaviors.

b: Local exposure to COVID-19 has a significant effect on donation behaviors.

### 2.2. The mediating role of emotions

A great volume of research has explored the effect of different emotions on donation behaviors (Dickert et al., 2011a; Aknin et al., 2012). The most prominent research includes studies exploring the effect of negative emotions on prosocial behaviors. Abundant research supports that negative emotions, including fear, stress, sadness, and anger, are positively correlated with donations (Ashar et al., 2016; Fajardo and Salerno, 2017; van Doorn et al., 2017; Heinrichs et al., 2018; O’Loughlin Banks and Raciti, 2018).

Under the context of the COVID-19 pandemic, the Terror Management Theory (TMT) might explain the motivational effect of negative emotions on donations. The TMT (Greenberg et al., 1986; Rosenblatt et al., 1989) states that to alleviate the anxiety brought by the awareness of death, humans tend to perform prosocially to increase personal worth (Greenberg et al., 2014; Pyszczynski et al., 2015; Ramkissoon, 2021; Wang, 2022). Based on TMT, past research has examined whether mortality salience can increase donation behaviors. Most of these studies came to a consistent result that participants in the mortality salience group donated significantly more money than participants in the control group (Jonas et al., 2002; Belmi and Pfeffer, 2016; Bruine de Bruin and Ulqinaku, 2021). However, most of the relevant research was conducted based on an experimental design, in which the participants in the experimental group were primed with death anxiety through different methods (Hirschberger et al., 2008). Only a few studies studied TMT using real-life events that elicited participants’ negative emotions rather than manipulated mortality salience (Black et al., 2020).

With its high mortality rate, the COVID-19 pandemic is a perfect setting to test the Terror Management Theory (TMT). Based on TMT, living under the impact of COVID-19 can trigger death anxiety, which in turn, can motivate people to make more donations to epidemic areas and cope with terror by increasing their sense of personal value (Jin and Ryu, 2021; Ramkissoon, 2021; Chew, 2022). Recent research has shown that personal exposure and local exposure to COVID-19 can increase anxiety, elicit grief, and elevate the pressure people feel, which further increases prosociality to buffer the negative emotions (Flesia et al., 2020; Liu et al., 2020; Ramkissoon, 2022a,b). Past research on donations has also shown that negative emotions, including anxiety, pain, and anger, can trigger prosocial behaviors, including donations, to alleviate these uncomfortable emotions (Lazarus, 1991; Bagozzi et al., 1999). Therefore, we inferred that negative emotions triggered by the COVID-19 pandemic could lead people to donate more (Bennett, 2015; Ramkissoon, 2020, 2022a,b).

Meanwhile, positive emotions have also been tested as a significant predictor of donation behaviors (Fiala and Noussair, 2017; Hudson et al., 2019). Positive emotions, including hope, can not only promote a sense of solidarity and connection to the beneficiaries but also empower people with a feeling that they can do a greater good to society through donation (Cryder et al., 2013; Paxton et al., 2020). In other words, positive emotions give people strength, inspiring them to donate more to improve the situation (Liang et al., 2016). Kayser et al. (2010) found that for low-cost helping behaviors, participants with negative and positive emotions were more likely to give help compared to participants with neutral emotions. Similarly, Aknin et al. (2012) also observed that general positive emotions and happiness could increase prosocial investment, including monetary donations. Based on these findings, we propose the following hypotheses:

H2a: The positive emotion is a significant mediator of the relationship between personal exposure to COVID-19 and donations.

H2b: The positive emotion is a significant mediator of the relationship between local exposure to COVID-19 and donations.

H3a: The negative emotion is a significant mediator of the relationship between personal exposure to COVID-19 and donations.

H3b: The negative emotion is a significant mediator of the relationship between local exposure to COVID-19 and donations.

### 2.3. The mediating role of risk perception

Risk perception is an individual’s psychological evaluation of the possibility and the impact of an unfavorable outcome (Sjöberg, 2000). Risk perception has two dimensions: emotion dimension (feelings) and cognitive dimension (analysis; Slovic et al., 2004; Slovic and Peters, 2006). The emotional dimension of risk perception refers to the worry and fear an individual has toward danger (Coleman, 1993; Lee et al., 2021). Past research has shown that during the COVID-19 pandemic, risk perception and negative emotions are positively correlated (Capone et al., 2021; Han et al., 2021), which was also found in our data. Since the mediating role of negative emotions has been examined in the previous section, this study focused on the cognitive dimensions.

The cognitive dimension of risk perception is associated with the possibility and the severity of consequences assessed from available information (Bonnet et al., 2012). Under the context of the COVID-19 pandemic, previous research has used items including the estimated length of the pandemic, the perceived severity of the disease, the perceived risk of getting it, and the estimated impact of the pandemic on the socio-economic situation to measure risk perception (Blanco et al., 2021). Considering studies on the risk perception of COVID-19, the majority of them focused on the effect of risk perception on promoting individuals’ health preventive measures (Wise et al., 2020; Shabu et al., 2021). Only a few studies explored the effect of risk perception on prosocial behaviors such as donation and found that the risk perception of the COVID-19 pandemic has a significant mediating effect on donations (Li W. Q. et al., 2021).

According to Han et al. (2021), risk perception of the COVID-19 pandemic has two components—health risk and financial risk. Health-related risk perception of COVID-19 consists of the perceived vulnerability of getting infected and the perceived severity of the pandemic (Li W. Q. et al., 2021). Exposure to COVID-19 would lead to increased awareness of the severity of the pandemic and the risk of being infected. This risk assessment will, in turn, lead people to perform actions that can reduce their risk of being infected, for instance, donating money to help the government fight against the pandemic. Similarly, Baber et al. (2022) found that people with a higher perceived risk of the COVID-19 pandemic made more financial contributions in crowdfunding. However, risk perception may not always motivate donations when considering the financial side. From a socio-economic perspective, Bruine de Bruin et al. (2020) studied the risk perception of people by measuring participants’ perceived risk of getting COVID-19, dying if getting it, getting quarantined, losing their jobs, and running out of money (Bruine de Bruin et al., 2020; Bruine de Bruin, 2021). Their results showed that although the perceived financial risk is lower than the perceived health risk, it could still become the economic motivator to decrease donation behaviors. Thus, risk perception of COVID-19 might have positive and negative effects on donations. Indeed, current research regarding the effect of risk perception on donations generated conflicting results. For example, Blanco et al. (2021) found that risk perception is not a significant predictor of donation during the COVID-19 pandemic, while Li W. Q. et al. (2021) concluded the opposite. The inconsistent results might be caused by using different measures for risk perception and mixing different components of risk perception in the studies. Given that financial risk has been found to decrease the willingness to donate, the present study only focused on the health component of risk perception, hoping to find a positive relationship between risk perception and donation behaviors. Thus, we derive the following hypotheses:

H4a: The risk perception is a significant mediator of the relationship between personal exposure to COVID-19 and donations.

H4b: The risk perception is a significant mediator of the relationship between local exposure to COVID-19 and donations.

### 2.4. The present study

Through the psychological framework we proposed above, the study connected the severity of the pandemic with its psychological consequences and used this connection to contribute to understanding factors that might affect donation behaviors during a global health emergency event. Although there are a few studies about donation behaviors during the COVID-19 pandemic, the amount of research on this topic is still lacking; our study adds to the literature in several ways. First, this study gave further texture to previous conclusions regarding the effect of exposure to COVID-19 by investigating personal exposure and local exposure separately. The individual experience with COVID-19 and the objective severity of the pandemic in the local community might cause different emotional responses to and rational evaluations of the pandemic, leading to different prosocial behaviors (Grimalda et al., 2021). By discerning the differences between individual experiences and the societal situation during the pandemic, our study would contribute to the explanation of previous inconsistent results regarding the effect of exposure to COVID-19 on donations. Second, our study extended the literature on the effect of emotions on donations during public health emergencies. Past research on the impact of the COVID-19 pandemic has mainly focused on the emotional consequences of the COVID-19 pandemic and possible solutions to tackle the negativity experienced during the pandemic (Flesia et al., 2020; Liu et al., 2020; Ramkissoon, 2022a). Yet, the consequent changes in prosocial behaviors due to emotions during the pandemic did not receive enough attention and lacked research. Third, our study aimed to reconcile previous conflicts on the effect of risk perception on donations. Previous research has arrived at opposite results in the effect of risk perception partly due to the use of different measurements of risk perception during the COVID-19 pandemic across studies (Blanco et al., 2021; Han et al., 2021; Li W. Q. et al., 2021). By only focusing on the health component of risk perception, we simplified the complexity of risk perception with the realization of its multifacetedness and raised caution regarding attempts to investigate risk perception as a single-sided concept.

## 3. Methods

### 3.1. Participants

Participants were recruited through convenience sampling from an online survey platform in China. A total of 8,720 participants finished the online survey. The age range of the participants is 18–70. The mean age is 28.91 (*SD* = 8.869). Among all the participants, 3,803 are females (43.6%). Participants are from 33 of the 34 provinces in China. Participants spanned across several income levels, education levels, and social status levels. The demographic characteristics of this sample are reported in Table 1. These demographic variables were controlled in all analyses.

**Table 1:** Demographic descriptive statistics of participants.

Variables	Mean/proportion	Min	Max	SD
Age	28.91	18	70	8.869
Gender				
Female	43.6%			
Male	56.4%			
Education level				
Elementary school and below	0.4%			
Middle school	3.9%			
High school	23.7%			
Some college	23.8%			
Bachelor’s degree	44.3%			
Master’s degree and above	4.0%			
Monthly income				
Below 3,000 RMB	23.6%			
3,001–7,000 RMB	55.1%			
7,001–15,000 RMB	18.3%			
15,001–50,000 RMB	2.5%			
Above 50,000 RMB	0.6%			
Subjective social level				
1 = “Lowest social level”	4.4%			
2	6.3%			
3	13.7%			
4	15.1%			
5	26.0%			
6	16.6%			
7	10.2%			
8	5.3%			
9	1.4%			
10 = “Highest social level”	1.0%			

### 3.2. Procedure and measures

#### 3.2.1. Survey

The data used in the study came from a nationwide survey by the Center of Social Psychology, Institute of Sociology, Chinese Academy of Social Science. The original survey was conducted online in China from March 20 to 30th, 2020. The survey included questions about the attention paid to the COVID-19 pandemic, emotions, perceived severity of COVID-19, perceived vulnerability of COVID-19, preventive health practices taken, perceived efforts spent by the government and public agencies to stop COVID-19, the access to COVID-19 related information, personal values regarding fate, difficulties encountered during the COVID-19 pandemic, personal exposure to COVID-19, and demographic information. For this current study, we only included the items that were relevant to the present study below. Participants received 5–6 RMB (equivalent to 0.90 US dollars) as compensation upon the completion of the survey. At the end of the survey, participants were given the opportunity to donate their compensation.

#### 3.2.2. Emotions

We asked participants to report their current emotions using a five-point scale (1 = Not at all, 5 = Very strong). Eight emotions were measured, including optimism, calm, worry, helplessness, fear, sadness, anger, and panic. From the independent *t*-sample test, we found that only optimism (*p* < 0.001), calm (*p* < 0.001), helplessness (*p* < 0.001), and fear (*p* < 0.001) were significantly different between people who decided to donate and not to donate. That is, worry, sadness, anger, and panic were not significantly related to donation behaviors in the context of COVID-19. Thus, we derived two emotion measures—positive and negative emotion—based on only optimism, calm, helplessness, and fear. We excluded the other four nonsignificant emotions because adding them might dilute the potential effect that helplessness and fear as two negative emotions have on donations. Although some studies concluded that sadness, anger, horror, and disgust were also significant predictors of donations, not every negative or positive emotion would effectively predict donations when the context changes. Indeed, in past research regarding the effect of emotions on donations, the specific emotions that have been proven as significant predictors of donations vary across the studies, which leads to inconsistent conclusions about the effect of positive and negative emotions (Bennett, 2015; Fiala and Noussair, 2017; Hudson et al., 2019; McGuire et al., 2020). For the present study, the positive emotion was calculated as the mean of optimism and calm, while the negative emotion was calculated as the mean of helplessness and fear. A higher score means a stronger emotion for both positive and negative emotions.

#### 3.2.3. Risk perception

We measured the risk perception of COVID-19 using items that measured the perceived severity of COVID-19 and the perceived vulnerability of COVID-19 in the survey. The specific items used in the questionnaire were “What do you think is the possibility you will get COVID-19?,” “What do you think is the possibility that COVID-19 can be cured if one is infected?,” “Considering the current situation, do you think you are safe from the spread of COVID-19?,” “What do you estimate for the possibility that COVID-19 would spread in your community?,” “What do you estimate for the possibility that the transmission of COVID-19 virus from abroad would trigger another outbreak in China?,” “Do you think COVID-19 would be more dangerous than SARS?,” “How do you predict the future of COVID-19 pandemic in 1 month?,” and “How long do you think the COVID-19 pandemic would last?.” Items are measured on a scale of either four or five. After performing the reliability test for the scale, we found that deleting items “Do you think COVID-19 would be more dangerous than SARS?” and “How long do you think the COVID-19 pandemic would last?” would largely improve the internal consistency of the risk perception scale. After removing these two items, we reached a Cronbach’s Alpha of 0.621. After rescaling all items to a four-point Likert scale, we calculated the risk perception of COVID-19 as the mean score of the remaining six items. A higher score means a higher risk perception of COVID-19.

#### 3.2.4. Personal exposure to COVID-19

The measure of personal exposure to COVID-19 is calculated as the mean of the three items in the survey, including the incidence of family members getting infected, the incidence of close friends getting infected, and the self-experience of quarantine. The incidence of family members getting infected is reported on a four-point scale (1 = family members got infected and recovered, 2 = family members got infected and are still under treatment, 3 = family members got infected and passed away, and 4 = none of the family members got infected). The incidence of close friends getting infected is reported on a four-point scale (1 = friends got infected and recovered, 2 = friends got infected and are still under treatment, 3 = friends got infected and passed away, and 4 = none of the friends got infected). The self-experience of quarantine is reported on a binary scale where 1 means the participants had the experience of quarantine and 0 means the participants did not have the experience of the quarantine. When calculating the personal exposure to COVID-19, we recoded “none of the family members got infected” and “none of the friends got infected” to 0 and rescaled the self-experience of quarantine to a scale of four. The higher the score, the more severely the person is exposed to COVID-19. The Cronbach’s Alpha for the three items of personal exposure is 0.508. Although this is a relatively low Cronbach’s Alpha, the low alpha is reasonable in the current context since the answers to the three items varied depending on each person’s experience with COVID-19.

#### 3.2.5. Local exposure to COVID-19

Local exposure is measured as the total number of new cases confirmed in the participant’s province 30 days prior to the participant’s response date. The data are obtained from The National Health Commission of the People’s Republic of China. Since the COVID-19 case data only included the provinces in Mainland China, we excluded three participants who resided in Hong Kong and Taiwan when calculating the effect of local exposure on donations (*N* = 8,717). The number of new confirmed cases in every province was not evenly distributed among all provinces. Some provinces had thousands of cases, while other provinces had nearly zero cases. For the accuracy of the data analysis, we calculated the log of the number of new confirmed cases to the base of *e* and used it in later statistical computation.

#### 3.2.6. Donation

At the end of the survey, the participants were asked if they would like to donate their compensation to public agencies to help fight against the pandemic. The participants also chose which agency they would like to donate to in the options. When calculating the donation measure, we considered all participants who chose to donate as a donator (coded as 1) regardless of the agency they chose to donate to. We coded participants who chose not to donate as 0.

## 4. Results

### 4.1. The descriptive statistics of the measures and the comparison between the donate and not donate group

For the whole sample, as shown in Table 2, the mean score of the positive emotion was 3.478, *SD* = 1.036. The mean score of the negative emotion was 2.156, *SD* = 1.077. The mean score of the risk perception was 1.917, *SD* = 0.397. The mean personal exposure was 0.372, *SD* = 0.421, indicating a relatively low personal exposure level for all participants. The mean local exposure was 2.872, *SD* = 1.872. Among all the participants, 15.4% of them chose to donate to a charity (*N* = 1,374), while 84.6% of them chose not to donate (*N* = 7,373). For the people who donated, the mean score of positive emotion was 3.602 (*SD* = 1.046), while the mean was 3.455 (*SD* = 1.032) for people who did not donate, which means participants who donated were more positive than participants who did not donate. The mean score of negative emotions was 2.297 (*SD* = 1.131) for people who donated and 2.130 (*SD* = 1.065) for people who did not donate, which means participants who donated also had stronger negative emotions compared with participants who did not donate. Overall, donors had stronger emotions than non-donors for both negative and positive emotions. The mean score of the risk perception was 1.990 (*SD* = 0.416) for people who donated and 1.904 (*SD* = 0.392) for people who did not donate, indicating donors had a higher risk perception. The mean personal exposure was 0.459 (*SD* = 0.521) for people who donated and 0.356 (*SD* = 0.398) for people who did not donate, indicating donors were affected by COVID-19 more personally than non-donors. The mean local exposure was 2.807 (*SD* = 1.828) for people who donated and 2.884 (*SD* = 1.880) for people who did not donate, which means the province where donors lived had fewer confirmed cases than the province where non-donors lived.

**Table 2:** Descriptive statistics of the measures.

Sample	Variables	*M*	SD
Full sample (*n* = 8,720)	Positive	3.478	1.036
	Negative	2.156	1.077
	Risk perception	1.917	0.397
	Personal exposure	0.372	0.421
	Local exposure	2.872	1.872
Donate (*n* = 1,347)	Positive	3.602	1.046
	Negative	2.297	1.131
	Risk perception	1.990	0.416
	Personal exposure	0.459	0.521
	Local exposure	2.807	1.828
Not donate (*n* = 7,373)	Positive	3.455	1.032
	Negative	2.130	1.065
	Risk perception	1.904	0.392
	Personal exposure	0.356	0.398
	Local exposure	2.884	1.880

### 4.2. The correlation analysis between examined variables and donation behaviors

We conducted a bivariate correlation analysis in SPSS using Spearman coefficient. The results show that the positive emotion (*r* = 0.054, *p* < 0.001), the negative emotion (*r* = 0.053, *p* < 0.001), the risk perception (*r* = 0.074, *p* < 0.001), and the personal exposure (*r* = 0.061, *p* < 0.001) are all positively correlated with donation behaviors (Table 3). Participants who donated had more positive and negative emotions, a higher risk perception, and a higher personal exposure level compared with participants who did not donate. The local exposure did not have a significant negative correlation with donation behaviors (*r* = −0.012, *p* = 0.250). These results supported our hypothesis H1a that personal exposure has a significant positive effect on donation behaviors but rejected our hypothesis H1b by showing that local exposure does not have a significant effect on donations.

**Table 3:** The correlation analysis between key variables.

Variables	1	2	3	4	5
1 Positive emotions	1.000				
2 Negative emotions	−0.312^***^	1.000			
3 Risk perception	−0.195^***^	0.314^***^	1.000		
4 Personal exposure	−0.006	−0.043^***^	0.105^***^	1.000	
5 Local exposure	0.008	0.36^***^	0.055	0.052^***^	1.00
6 Donations	0.054^***^	0.053^***^	0.074^***^	0.061^***^	−0.012

^*^p < 0.05, ^**^p < 0.01, and ^***^p < 0.001.

### 4.3. The mediation models

We used Mplus to examine the mediation models in this study. For all the models, 40,000 biased-corrected bootstrap samples were used to create 95% confidence intervals.

#### 4.3.1. The mediation model between personal exposure and donations with negative and positive emotions and risk perception as the mediators

**Figure 2:**
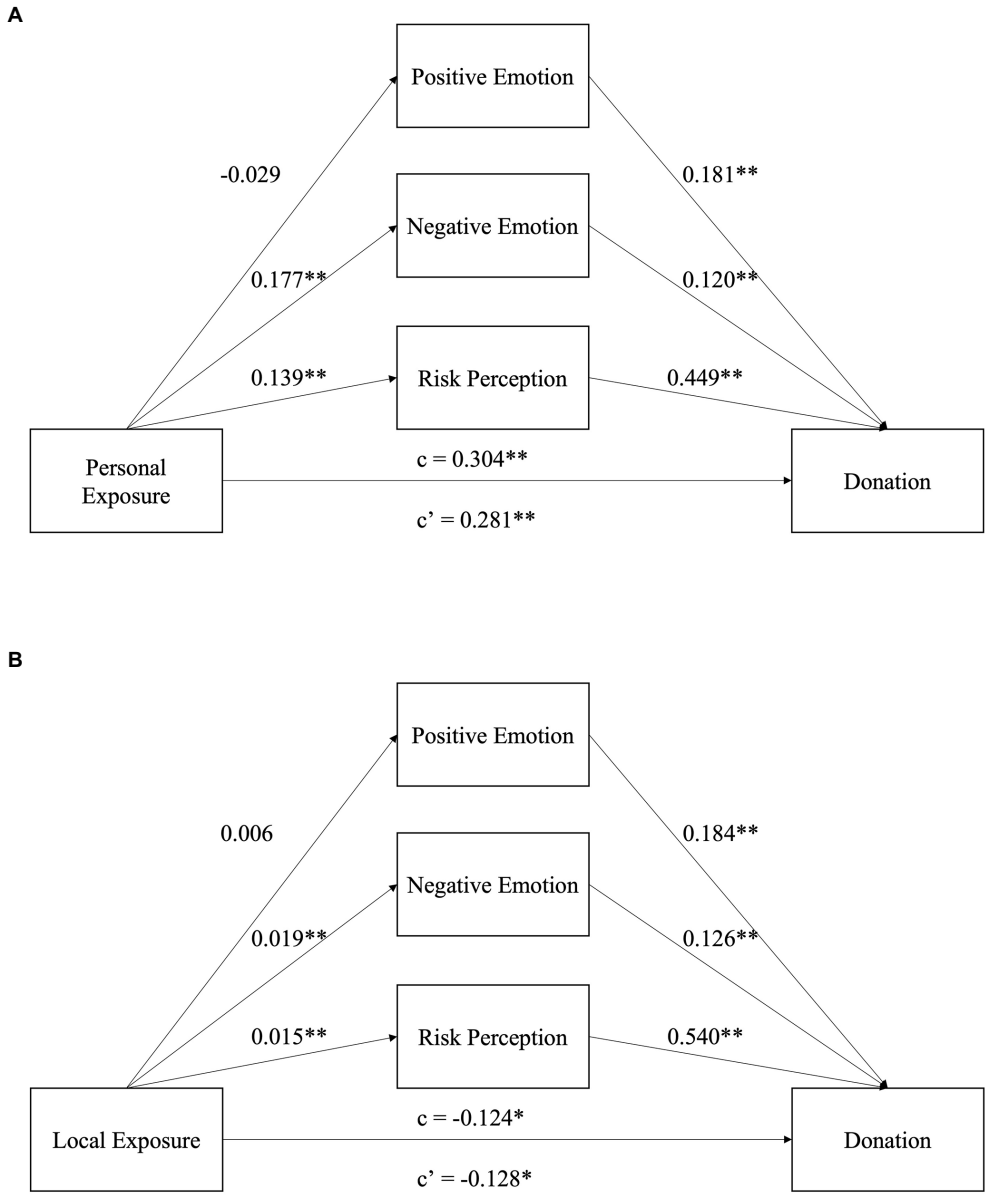
The mediation model of the relationships between personal exposure and donation and between local exposure and donation. **(A)** The mediation model of personal exposure with positive emotion, negative emotion, and risk perception as mediators. **(B)** The mediation model of local exposure with positive emotion, negative emotion, and risk perception as mediators. ^*^p < 0.05, ^**^p < 0.01. c, total effect; c’, direct effect.

We combined the three mediators into one paralleled mediation model for the relationship between personal exposure and donations. Based on our results (Table 4), the three mediators combined had a significant mediating effect on the relationship between personal exposure and donations (*ab* = 0.023, *p* < 0.001, *CI* = [0.015, 0.033]). The results show that higher personal exposure (*b* = 0.407, *p* < 0.001, *CI* = [0.271, 0.539]), stronger positive emotions (*b* = 0.181, *p* < 0.001, *CI* = [0.118, 0.246]), stronger negative emotions (*b* = 0.120, *p* < 0.001, *CI* = [0.063, 0.176]), and higher risk perception (*b* = 0.449, *p* < 0.001, *CI* = [0.290, 0.611]) all predicted more donation behaviors (Table 4). However, personal exposure was not significantly associated with positive emotions (*a* = −0.029, *p* = 0.259, *CI* = [−0.079, 0.021]). Positive emotions did not have a significant mediating effect on the relationship between personal exposure and donation behaviors (*ab* = −0.003, *p* = 0.278, *CI* = [−0.009, 0.002]). Thus, positive emotions were not a mediator for the positive relationship between personal exposure and donations, rejecting our hypothesis H2a. On the contrary, personal exposure had a significant positive relationship with negative emotions (*a* = 0.177, *p* < 0.001, *CI* = [0.123, 0.229]) and risk perception (*a* = 0.139, *p* < 0.001, *CI* = [0.119, 0.158]). Negative emotions (*ab* = 0.013, *p* < 0.001, *CI* = [0.006, 0.021]) and risk perception (*ab* = 0.013, *p* < 0.001, *CI* = [0.009, 0.019]) had a significant mediating effect on the relationship between personal exposure and donation behaviors, which supported our hypotheses H3a and H4a. The direct effect of personal exposure on donations after mediation was 0.281 (*p* < 0.001, *CI* = [0.188, 0.370]). The total effect was 0.304 (*p* < 0.001, *CI* = [0.211, 0.393]). Thus, higher personal exposure led to higher negative emotions and higher risk perception, which in turn led to donation behaviors. This model is illustrated in Figure 2A.

**Table 4:** The mediation analysis on the relationship between personal exposure and donation behavior with positive emotions, negative emotions, and risk perceptions as mediators.

Path	Estimate	S.E.	95%CI	*p* value
Personal exposure → Positive emotions	−0.029	0.026	[−0.079, 0.021]	0.259
Personal exposure → Negative emotions	0.177	0.027	[0.123, 0.229]	<0.001^***^
Personal exposure → Risk perception	0.139	0.010	[0.119, 0.158]	<0.001^***^
Positve emotions → Donation	0.181	0.033	[0.118, 0.246]	<0.001^***^
Negative emotions → Donation	0.120	0.029	[0.063, 0.176]	<0.001^***^
Risk perception → Donation	0.449	0.082	[0.290, 0.611]	<0.001^***^
Personal exposure → Donation	0.407	0.069	[0.271, 0.539]	<0.001^***^
Personal exposure → Positive emotions → Donation	−0.003	0.003	[−0.009, 0.002]	0.278
Personal exposure → Negative emotions → Donation	0.013	0.004	[0.006, 0.021]	<0.001^***^
Personal exposure → Risk perception → Donation	0.013	0.003	[0.009, 0.019]	< 0.001^***^
Direct effect after mediation	0.281	0.047	[0.188, 0.370]	<0.001^***^
Indirect effect	0.023	0.005	[0.015, 0.033]	<0.001^***^
Total effect	0.304	0.047	[0.211, 0.393]	<0.001^***^

^*^p < 0.05, ^**^p < 0.01, and ^***^p < 0.001.

#### 4.3.2. The mediation model between local exposure and donations with negative and positive emotions and risk perception as mediators

Our results (Table 5) showed that the three mediators combined had a significant mediating effect on the relationship between local exposure and donations (*ab* = 0.004, *p* < 0.001, *CI* = [0.002, 0.006]). The results show that positive emotions (*b* = 0.184, *p* < 0.001, *CI* = [0.121, 0.249]), negative emotions (*b* = 0.126, *p* < 0.001, *CI* = [0.070, 0.182]), and risk perception (*b* = 0.540, *p* < 0.001, *CI* = [0.383, 0.700]) were significantly positively associated with donation behaviors, whereas local exposure was significantly negatively associated with donation behaviors (*b* = −0.042, *p* = 0.012, *CI* = [−0.074, −0.009]). In addition, local exposure had a significant positive relationship with negative emotions (*a* = 0.019, *p* = 0.003, *CI* = [0.007, 0.031]) and risk perceptions (*a* = 0.015, *p* < 0.001, *CI* = [0.010, 0.019]) but not with positive emotions (*a* = 0.006, *p* = 0.335, *CI* = [−0.006, 0.017]). Positive emotions did not have a significant mediating effect on the relationship between local exposure and donation behaviors (*ab* = 0.001, *p* = 0.351, *CI* = [−0.001, 0.002]). Thus, positive emotions were not a mediator for the positive relationship between local exposure and donations, rejecting our hypothesis H2b. On the contrary, negative emotions (*ab* = 0.001, *p* = 0.014, *CI* = [0.001, 0.003]) and risk perception (*ab* = 0.002, *p* < 0.001, *CI* = [0.001, 0.003]) had a significant mediating effect on the relationship between local exposure and donation behaviors. However, since the local exposure was negatively associated with donation, we had a negative direct effect of local exposure on donations after mediation is −0.128 (*p* = 0.012, *CI* = [−0.228, −0.029]). The total effect was −0.124 (*p* = 0.014, *CI* = [−0.224, −0.025]). Thus, although, overall, higher local exposure directly led to fewer donations, it also led to a higher level of negative emotions and risk perception, which in turn indirectly led to more donation behaviors. Our hypotheses H3b and H4b are partially supported. This model is illustrated in Figure 2B.

**Table 5:** The mediation analysis on the relationship between local exposure and donation behavior with positive emotions, negative emotions, and risk perceptions as mediators.

Path	Estimate	S.E.	95%CI	*p* value
Local exposure → Positive emotions	0.006	0.006	[−0.006, 0.017]	0.335
Local exposure → Negative emotions	0.019	0.006	[0.007, 0.031]	0.003^**^
Local exposure → Risk perception	0.015	0.002	[0.010, 0.019]	<0.001^***^
Positve emotions → Donation	0.184	0.033	[0.121, 0.249]	<0.001^***^
Negative emotions → Donation	0.126	0.029	[0.070, 0.182]	<0.001^***^
Risk perception → Donation	0.540	0.081	[0.383, 0.700]	<0.001^***^
Local exposure → Donation	−0.042	0.017	[−0.074, −0.009]	0.012^*^
Local exposure → Positive emotions → Donation	0.001	0.001	[−0.001, 0.002]	0.351
Local exposure → Negative emotions → Donation	0.001	0.001	[0.001, 0.003]	0.014^*^
Local exposure → Risk perception → Donation	0.002	0.000	[0.001, 0.003]	< 0.001^***^
Direct effect after mediation	−0.128	0.051	[−0.228, −0.029]	0.012^*^
Indirect effect	0.004	0.001	[0.002, 0.006]	<0.001^***^
Total effect	−0.124	0.051	[−0.224, −0.025]	0.014^*^

^*^p < 0.05, ^**^p < 0.01, and ^***^p < 0.001.

## 5. Discussion

Humans are paradoxical entities in that we present selfishness and altruism at the same time when facing a collective threat (Fridman et al., 2022). How to maximize generosity and minimize selfishness among people and communities under uncertainties has always been an interesting topic that has important implications in the realm of social psychology. This study sought to understand the determinants of people’s donation behavior under the impact of public emergencies. Our study showed that positive emotions, negative emotions, risk perception, and personal exposure were positively correlated with donation behaviors. To explore the interaction between these factors, we conducted the mediation analysis and generated the following important findings and conclusions.

First, in the context of public health emergencies like the COVID-19 pandemic, negative emotion is a major determinant that promotes donation. Negative emotions mediated the relationships between donations and exposure to COVID-19, both personally and locally. Specifically, the higher personal and local exposure led to a stronger negative emotion, which in turn, led to donation behaviors. This result aligns with findings that COVID-19-induced fear leads to greater monetary donations from Jin and Ryu (2021) and Chew (2022), whose research also investigates the effect of negative emotions on prosocial behaviors under the Terror Management Theory. Following the TMT, the helplessness and the fear of getting infected by COVID-19 drove people to donate more by perceiving the donation behaviors as actions praised by their cultural values and self-esteem, which works as a psychological buffer to relieve the uncomfortable feelings triggered by close experience with the pandemic. The emotional buffer provided by donation behaviors might be the reason behind the mediating effect of negative emotions on donations. Moreover, we did realize that the negative emotions—helplessness and fear—used in our study might not always be the effective negative emotions that promote donations since emotions are context-specific. Caution is needed when generalizing our conclusion to future research when the context changes.

Second, although positive emotion is a general determinant of donation, it is not a major factor that leads to donations under the influence of public emergencies. In line with Liang et al. (2016), Bae (2021), and Bennett (2015), our results showed that both positive emotions and negative emotions were significant predictors of donations. However, our study also indicated that the negative emotion but not the positive emotion was a significant mediator of the relationship between exposure to COVID-19 and donations, which introduced a unique angle that has not been explored in the field. A possible explanation for this result could be that, under higher exposure to COVID-19, people tend to donate more to alleviate their negative emotions, including fear and anxiety toward the pandemic, which follows the Terror Management Theory. In contrast, positive emotions may be triggered by seeing optimistic updates on the pandemic, including news on government measurement, which is a different pathway from personal exposure and local exposure to COVID-19. Through positive emotions, these promising updates gave people strength and the belief that the situation would be better with their help, which promoted donation willingness (Liang et al., 2016; Paxton et al., 2020). The different effects of positive and negative emotions found in our study add further color to the current literature on donations during the COVID-19 pandemic.

Third, risk perception is also a major determinant of donation behaviors under public health emergencies. The risk perception mediated the relationship between donations and both personal and local exposure. This mediating effect can be explained by the following: The higher exposure to COVID-19 provides more information for risk analysis and leads people to assess the current situation as less favorable, thus increasing their risk perception of the pandemic. The increased risk perception reminds people of their vulnerability to the virus, which in turn, motivates donation behaviors. Our finding is in line with finding of Li W. Q. et al. (2021) while is opposite to finding of Blanco et al. (2021). The use of different concepts and different measures for risk perception could contribute to contradictory results. For example, Blanco et al. (2021) used a set of questions, including both health-related items and financial-related items, to measure risk perception. In contrast, Li W. Q. et al. (2021) only used health-related items—perceived vulnerability and perceived severity—to measure risk perception. In the end, they reached the opposite results: Blanco et al. found no significance for the effect of risk perception on donations, while Li W. Q. et al. (2021) found significance for it. Similar to Li W. Q. et al. (2021), we only use health-related items to measure risk perception, which might explain the same results we had with Li et al.’s study. As Han et al. (2021) stated that risk perception is composed of health risk and financial risk, together with Blanco et al.’s and Li et al.’s results, our results showed that health risk and financial risk perception have different influences on people’s donation behaviors and should be studied separately in future studies.

Fourth, personal exposure to COVID-19 is the most direct environmental factor of donation promotion. Paralleling with prior studies on donation under public emergencies (Grimalda et al., 2021), our study showed that under the impact of COVID-19, people would be more likely to donate if they were exposed to COVID-19 cases personally but not locally. The higher level of personal exposure not only suggests a higher risk of infection for the participants but also heavily impacts their emotions. These cognitive and emotional responses to collective threats further drive people to donate more. However, local exposure in the form of an objective number could not induce the same level of perceived risks and negative emotions comparable to personal exposure, which might explain the nonsignificant direct effect of local exposure versus the significant effect of personal exposure.

Fifth, although local exposure to COVID-19 is an environmental factor that can promote donations, its effect on donations is not a direct one. From the correlation analysis, local exposure had a negative relationship with donations, but this relationship was not significant, which corresponds with the null effect of local exposure found in studies of Zheng et al. (2021a), Grimalda et al. (2021), and Brañas-Garza et al. (2022). However, from the mediation analysis, local exposure did indirectly increase donation behaviors through negative emotions and risk perception. The reason behind this result could be other characteristics of the local community, for example, economics, urbanization, and cultural differences, which had a negative influence on donation willingness and reduced the positive effect on local exposure to COVID-19 as a whole. For example, if a local community is under an individualistic culture, it would decrease people’s willingness to donate (Zheng et al., 2021a). Similarly, as Adena and Harke (2022) found in their research, after controlling all of the local economic characteristics, local pandemic severity has a positive effect on donations to COVID-19-related charities in the United Kingdom. The unexpected results of local exposure in our study emphasized the complexity of local exposure as a determinant of prosocial behaviors. Future research is needed to unravel the factors of local exposure that moderate its effect.

To sum up, negative emotions and risk perception are two major determinants of donation behaviors in uncertain social contexts like COVID-19. Personal exposure and local exposure to public health emergencies are two major external environmental factors that affect donation. However, personal exposure promote donation directly, while local exposure promotes donation indirectly through the mediated effects of negative emotions and risk perception.

### 5.1. Theoretical contributions and practical implications

Our study has several contributions to the literature and implications for the real-world dilemma. First, our study proposed a psychological framework that promotes donations under the uncertainty brought by public health emergencies such as the COVID-19 pandemic. Under this theoretical framework, we examined donation behaviors based on survey data analysis and found that negative emotion and risk perception are two major factors that influenced donation willingness under public health emergencies. Among the few studies investigating public emergencies, most of them focused on natural disasters such as hurricanes, which only affect a relatively small number of people in a restricted area. Focusing on COVID-19, our study complemented the lack of research on public health emergencies, a research setting that urgently deserves more research due to its global transmissibility and impact on different social aspects.

Second, using the COVID-19 pandemic as a natural testing environment, our results provided evidence for the effectiveness of Terror Management Theory in real life. TMT has been extensively studied under experimental controlled settings. However, little was known about how people would behave in the context of real-life events. Our study extended the current literature on the Terror-Management Theory by using it to explain donation behaviors during the COVID-19 pandemic. According to TMT, death thoughts can motivate people to perform prosocial behaviors to alleviate anxiety. Negative emotions induced by the health risk and the threat of death under the COVID-19 pandemic led people to make more donations in order to get rid of this unpleasant feeling. In addition, most past research on TMT was conducted in western countries with individualistic cultures. Using the data from China, our study supported the consistency of TMT in collectivist cultures as well.

Third, by introducing the concept of risk perception to donation motivation research, we separated the emotional dimension and the cognitive dimension of risk perception and explored the effect of the cognitive dimension on donations. On the one hand, our study enriched the application of risk perception theory by introducing it as a mechanism that motivates prosocial behaviors under collective threats. On the other hand, we expanded the psychological mechanism of donation behaviors. Past research on the determinants of donations primarily focused on the emotional aspect of risk perception, whereas research on the effect of cognitive aspects on donations is still lacking. Besides the fear of getting COVID-19, we emphasized the importance of the cognitive assessment of the risk related to COVID-19 as a motivation to donate. Moreover, we also attempted to solve the conflicting results of the effect of risk perception in COVID-19-related literature by separating health risks and financial risks. Our results provided a more nuanced assertion that health-related risk perception is positively correlated with donation behaviors. The effect of financial risk on donations still needs future research.

Fourth, we categorized exposure to COVID-19 into personal exposure and local exposure and examined the effect of these two kinds of exposure separately, which distinguishes our study from prior studies. Specifically for local exposure, we did not naively conclude that it was not associated with donation behaviors. Instead, by analyzing the mediation effects, we found that local exposure indirectly promoted donations. Through this method, we prevented the possibility that the side effects of some local characteristics, including economic, urbanization, and cultural differences, diminish the effect of the severity of the local pandemic on donations. More importantly, by separating personal exposure and local exposure from each other, we offered a new perspective on analyzing the effect of exposure to the pandemic on donations and demonstrated that these two kinds of exposure have different psychological impacts on people facing a collective threat.

Lastly, our study enriched the research on emotions involved in donations in several ways. The research on the effect of emotions on donation behaviors has generated opposite results among different studies (Bennett, 2015; Fiala and Noussair, 2017; Best and Costello, 2019). With the hope of reconciling these conflicts, our results supported some of the past research and opposed the rest, providing more evidence to this debate. Besides, most research investigating the effect of emotions on donation behaviors focused on the emotional appeals of the advertisement rather than the general mood of the donors themselves. Furthermore, emotion research in the context of COVID-19 has not paid enough attention to the consequent social behaviors led by emotions. Our research filled these research gaps by stating that negative emotion is a determinant of donation during the COVID-19 pandemic.

As for the implications for practice, our study provided several suggestions on how to raise more donations when fighting against public emergencies. First, our study found that under higher exposure to the pandemic, people are more likely to donate. Although it would be unethical to raise money in impacted areas and from people who suffered from COVID-19, we could generate a feeling of personally experiencing COVID-19 for people who are unaffected by the pandemic through publicizing stories of affected families and doctors in the front line. Second, our results demonstrated that people are more likely to donate when they are in a higher emotional state. Thus, the government should expose the situation in the impacted area more on media to elicit anxiety and fear while reporting the current effort public agencies make to give people hope at the same time. Charity organizations should also consider increasing their donation marketing when people have the strongest emotional response to emergency events, for instance, near the beginning of the emergency. Third, since the study showed that during public health emergencies, people with higher risk perception are more likely to donate, the media can consider emphasizing the severity of the pandemic to induce awareness of vulnerability to disease.

### 5.2. Limitations and future research

We found several limitations of our study and derived suggestions for future research from them. First, our study measured the donation behaviors by participant’s donation choice at the end of the survey. However, the donation made in our study did not represent the overall donation behaviors of the participants during the COVID-19 pandemic. Participants might donate to other charitable organizations through different platforms on their own. Since we investigated the general characteristics of the donors during the pandemic, the overall actual donations participants made to public agencies might be a better measurement to assess their donation behaviors. For future research, a self-report of donation behaviors and the data from the donation database could be added to the measurement for a more accurate result.

Second, the severity of the COVID-19 pandemic highly interfered with our results. During the time for data collection, the severity of the pandemic in China has already been mitigated, with single-digit new confirmed cases daily. Based on our results and prior studies (Grimalda et al., 2021; Zheng et al., 2021a), the severity and exposure to the COVID-19 pandemic will significantly influence donation behaviors. A less severe situation will lead to fewer donations. Indeed, in our study, only 15.4% of the participants donated their compensation. In Grimalda et al. (2021) study conducted during the first wave of the pandemic, 63% of the participants in the US and 77% of the participants in Italy chose to donate. For future research on public emergencies, the study should start as soon as the onset of the disaster. With higher severity, more donations would be made, which could lead to more significant results.

Third, although risk perception theory was proposed long ago, there is no standard measurement for risk perception, which might lead to contradictory results. If two studies focusing on risk perception used different sets of items to measure it, then the possibility for them to generate different results for the same research question is high since the measurements used in the analysis are essentially different concepts. In order to eliminate the possible error of measurement, future research could design a standard questionnaire to assess risk perception on which items should be interchangeable for different situations.

Lastly, the statistical analysis method we used in our study is still immature. The standard statistical analysis method for analyzing the mediation effect of categorical dependent variables has not been developed in the field. We used the statistical analysis method Fang et al. (2017) proposed in their study and calculated the mediation models in MPlus. However, the mediation analysis method developed by Fang et al. has not yet been recognized as the standard method for calculating the mediation effect of the categorical dependent variable. Future research is needed to develop a standard statistical analysis method for the mediation of categorical dependent variables.

## Data availability statement

The raw data supporting the conclusions of this article will be made available by the authors, without undue reservation.

## Ethics statement

The studies involving human participants were reviewed and approved by Institute of Sociology, Chinese Academy of Social Sciences. The patients/participants provided their written informed consent to participate in this study.

## Author contributions

YZ carried out the experiment. YZ and YB conceived the original idea. YB performed computation on the data and wrote the manuscript with support from YZ and JW. JW supervised the project. All authors contributed to the article and approved the submitted version.

## Funding

The study was funded by Key Projects of Philosophy and Social Sciences Research, Ministry of Education (Grant Number is 21JZD038).

## Conflict of interest

The authors declare that the research was conducted in the absence of any commercial or financial relationships that could be construed as a potential conflict of interest.

## Publisher’s note

All claims expressed in this article are solely those of the authors and do not necessarily represent those of their affiliated organizations, or those of the publisher, the editors and the reviewers. Any product that may be evaluated in this article, or claim that may be made by its manufacturer, is not guaranteed or endorsed by the publisher.
